# NLRP6 negatively regulates host defense against polymicrobial sepsis

**DOI:** 10.3389/fimmu.2024.1248907

**Published:** 2024-04-23

**Authors:** Laxman Ghimire, Sagar Paudel, John Le, Liliang Jin, Shanshan Cai, Dinesh Bhattarai, Samithamby Jeyaseelan

**Affiliations:** ^1^ Laboratory of Lung Biology, Department of Pathobiological Sciences and Center for Lung Biology and Disease, School of Veterinary Medicine, Louisiana State University (LSU) and Agricultural and Mechanical College, Baton Rouge, LA, United States; ^2^ Section of Pulmonary and Critical Care, Department of Medicine, LSU Health Sciences Center, New Orleans, LA, United States

**Keywords:** NLRP6, neutrophil, cytokine, chemokine, innate immunity

## Abstract

**Introduction:**

Sepsis remains a major cause of death in Intensive Care Units. Sepsis is a life-threatening multi-organ dysfunction caused by a dysregulated systemic inflammatory response. Pattern recognition receptors, such as TLRs and NLRs contribute to innate immune responses. Upon activation, some NLRs form multimeric protein complexes in the cytoplasm termed “inflammasomes” which induce gasdermin d-mediated pyroptotic cell death and the release of mature forms of IL-1β and IL-18. The NLRP6 inflammasome is documented to be both a positive and a negative regulator of host defense in distinct infectious diseases. However, the role of NLRP6 in polymicrobial sepsis remains elusive.

**Methods:**

We have used NLRP6 KO mice and human septic spleen samples to examine the role of NLRP6 in host defense in sepsis.

**Results:**

NLRP6 KO mice display enhanced survival, reduced bacterial burden in the organs, and reduced cytokine/chemokine production. Co-housed WT and KO mice following sepsis show decreased bacterial burden in the KO mice as observed in singly housed groups. NLRP6 is upregulated in CD3, CD4, and CD8 cells of septic patients and septic mice. The KO mice showed a higher number of CD3, CD4, and CD8 positive T cell subsets and reduced T cell death in the spleen following sepsis. Furthermore, administration of recombinant IL-18, but not IL-1β, elicited excessive inflammation and reversed the survival advantages observed in NLRP6 KO mice.

**Conclusion:**

These results unveil NLRP6 as a negative regulator of host defense during sepsis and offer novel insights for the development of new treatment strategies for sepsis.

## Introduction

Sepsis is a leading cause of death in intensive care units (ICU) (1) and the most expensive disease as it accounted for more than $20 billion in hospital expenses in 2011 (2, 3). The third consensus conference has defined sepsis as “a life-threatening organ dysfunction caused by a dysregulated host response to infection” (1, 4). The estimated annual incidence of sepsis is around 19 million worldwide (5) and the incidence is increasing due to increased number of immunocompromised patients and improvement in identification of septic patients (1). Numerous clinical trials have been performed in past 20 years; however, none of these trials have succeeded in providing an effective drug that could be used to treat sepsis patients (4). In this situation, research to understand the detailed pathophysiology of sepsis is warranted to identify potential new/specific drug targets for treatment.

Sepsis is a multi-organ disease caused by excessive innate immune response to microbial infection. Pattern recognition receptors like Toll-Like Receptors (TLRs) and Nod-Like Receptors (NLRs) are germline-encoded innate immune components which play critical roles in initiating the innate immune response to microbial infection. Unlike TLRs, certain NLRs have the ability to bind with an adaptor molecule like ASC (Apoptosis-associated speck-like protein containing CARD) and recruit an enzyme called caspase-1 to form a multiprotein complex known as an inflammasome (6). The active caspase-1 cleaves the inactive form of IL-1β and IL-18 to their active form to initiate an effective innate immune response (6, 7). While the roles of TLRs, including TLR2, TLR3, TLR4, and TLR9 (8–11) in the outcome of sepsis are well understood, a limited number of studies (12, 13) have focused on determining the roles of NLRs in the pathophysiology of sepsis.

NLRP6 is a relatively new member of NLR family which is shown to form an inflammasome during both infectious (14, 15) and non-infectious inflammation (16). The NLRP6 inflammasome has been reported as a positive and negative regulator of host defense (17). Using a mouse model of pulmonary *Staphylococcus aureus* infection, the NLRP6 inflammasome was found to negatively regulate neutrophil-dependent host immunity (14). Similar negative roles of NLRP6 were also reported during other bacterial infections such as *Salmonella typhimurium*, *Streptococcus pneumoniae*, and *Listeria monocytogenes* (15, 18, 19). On the other hand, a study using an enteric pathogen, *Citrobacter rodentium*, the NLRP6 inflammasome was found to be important for host defense as a positive regulator (20). Additionally, during viral infection, NLRP6 KO mice showed increased viral burden in the intestine compared to their WT counterparts (21). In a similar manner, we previously reported that NLRP6 is essential for neutrophil-dependent host defense during pulmonary *Klebsiella pneumoniae* infection (22). These observations suggest that the function of the NLRP6 inflammasome could be model- or pathogen- specific. Although NLRP6 is well expressed in several organs including intestine, kidney, liver, and lung (23), its potential roles in sepsis remain elusive.

Here, we have used human septic spleen samples and a murine model of cecal ligation and puncture (CLP)-induced polymicrobial sepsis to study the detailed mechanisms by which NLRP6 regulates host defense. Using CLP, we demonstrate that the NLRP6 inflammasome plays a detrimental role following sepsis. Co-housed WT and NLRP6 KO mice following sepsis show decreased bacterial burden in KO mice similar to without co-housing. Using spleen samples from humans, we demonstrated upregulation of NLRP6 in multiple cells, including CD4 and CD8 T cells. The NLRP6 KO mice showed higher numbers of CD3-, CD4- and CD8-positive T cell subsets and decreased T cell death in the spleen following sepsis. In addition, CD8 T cells but not CD4 T cells mediate bacterial clearance following sepsis. NLRP6-driven IL-18 elicits hyperinflammation and administration of recombinant IL-18 reversed the survival advantage seen in NLRP6 KO mice. Taken together, our data implicated that the NLRP6 inflammasome serves as a negative regulator of host protection in sepsis. An improved understanding of the pathogenesis of sepsis will help design better therapeutic strategies to improve the disease outcome.

## Materials and methods

### Animals

C57BL/6 (wild-type, WT) mice were purchased from Jackson Laboratories (Bar Harbor, ME) whereas NLRP6 KO mice were obtained from Millennium Pharmaceuticals (Cambridge, MA). All these mice were on a C57BL/6 background and used at 8-10 weeks old. Animals were kept in specific pathogen free environments with access to food and water. All experiments were performed in accordance with the Guide for the Care and Use of Laboratory Animals of the National Institute of Health. This study was approved by Institutional Animal Care and Use Committee (IACUC) at Louisiana State University and Agricultural and Mechanical College in Baton Rouge, LA.

### Human tissue samples

De-identified human septic and non-septic spleen blocks (septic and non-septic) were kindly provided by Dr. Richard S Hotchkiss, Washington University School of Medicine at St. Louis, MO.

### Induction of sepsis

Polymicrobial sepsis was induced in mice using CLP as described in our previous publication (13). For bacterial sepsis, we injected *E. coli* at the dose rate of 5 X 10^8^ CFU/kg of mouse intra-peritoneally and observed survival for 12 days. Recombinant IL-18 was administered immediately after CLP (5 ug/mouse, IP) (24). CD4 or CD8 T cells were depleted by treating each mouse with 200 µg of anti-CD4 or anti-CD8 antibody (BioXcell, NH) 12 and 2 hours prior to CLP.

### Collection of peritoneal lavage fluid

After specific time points, mice were euthanized. The peritoneal cavity was washed by injecting 7 ml of PBS containing heparin and dextrose. The peritoneal lavage fluid (PF) was collected in a sterile conical tube. A total of 6ml of lavage fluid was collected from each mouse. The total and differential leukocyte count was performed in the peritoneal fluid as described in our previous publication (14).

### Flow cytometry

Flow cytometric staining was performed as recommended by the manufacturer’s protocol (BioLegend, San Diego, CA). The single cell suspensions obtained from septic WT and NLRP6 KO mice were stained with antibody against CD3ϵ (145-2C11), CD4 (GK1.5), CD8α (53-6.7), and PI (Thermo Fisher Scientific, Waltham, MA). The stained cells were fixed with 2% paraformaldehyde and analyzed using either a FACs Caliber or LSRFortessa X20 (BD). Appropriate isotype antibodies were used for each color. The data obtained were analyzed using FlowJo_V10 (Treestar).

### Immunofluorescence microscopy

Parafilm-embedded splenic tissue blocks from septic and non-septic patients were provided by Dr. Richard S. Hotchkiss, Washington University School of Medicine at St. Louis, MO. Immunofluorescence microscopy was performed in spleen tissue sections from human patients and mice as described in our previous publications (14, 25, 26). The following primary antibodies were used: human NLRP6, CD3, CD4, CD8 and mouse NLRP6, CD3, CD4, and CD8. After overnight incubation with these primary antibodies, the excess antibodies were washed off with cold PBS. The slides were then stained with appropriate secondary antibodies against human and mouse antigens for 30 minutes at room temperature. The image was taken under florescent microscope (Zeiss Axioskop 2 Plus) microscope.

### Cytokine measurement

Cytokines were measured using standard ELISA procedure as described in the manufacturer’s protocol (eBioscience or ThermoFisher scientific, MA).

### Cell death assay

Measurement of cell death was performed using Cytox-One Homogenous Membrane Integrity Assay Kit (Promega, WI) following the manufacturer’s protocol. LDH release was expressed in relative fluorescence units (RFU).

### Statistics

Data are presented as Mean ± SEM. Unpaired two-tailed student’s t test was used to compare the data between two groups. One-way ANOVA followed by Tukey’s multiple comparisons test was used whenever there were more than two groups. Statistical analysis was accomplished by GraphPad Prism 8.0 software (San Diego, CA). Survival analysis was performed using log-rank test. The experiments are representative of 2-3 independent experiments. A *p* value less than 0.05 was considered significant.

## Results

### NLRP6 impairs host protection following polymicrobial sepsis

**Figure 1:**
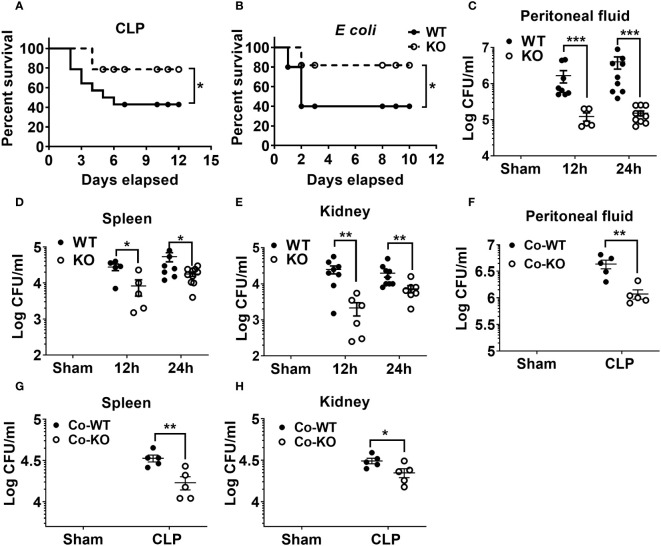
The impact of NLRP6 in host protection following CLP-induced sepsis. **(A)** C57BL/6 wild-type (WT) and NLRP6 KO mice (N=14 per group) were subjected to CLP-induced sepsis and survival was monitored for 12 consecutive days. Data were analyzed using Log-rank (Mantel-Cox) test (*p<0.05). **(B)** WT and NLRP6 KO mice (N=10 per group) were injected with 10^7^ CFUs of *E coli* per mouse intraperitoneally (IP) to induce bacterial sepsis and monitored for 12 days post-infection (N=10 per group each time). Log-rank (Mantel-Cox) test was used to analyze the data (*p<0.05). **(C-E)**. WT and KO mice (N=6-10 per group) were subjected to CLP-induced sepsis. Sham operated animals were used as control. At 12- and 24-hours post infection, mice were sacrificed to collect peritoneal lavage fluid (PF), spleen, and kidneys for estimating bacterial burden. Data were analyzed using unpaired two-tailed student’s *t* test for each time point (*p<0.05, **p<0.01, and ***p<0.001). **(F-H)** WT and NLRP6 KO mice (N=5 per group) were co-housed for 4 weeks and sepsis was induced via CLP. Twenty-four hours post-CLP, mice were euthanized to enumerate bacterial burden in PF, spleen, and kidneys. Data is represented as Mean ± SEM.

Our previous investigation demonstrated that NLRP6 serves as a negative regulator of host defense for *Staphylococcus aureus*-induced lung infection^12^ whereas it acts as a positive regulator of host immunity for pulmonary *K. pneumoniae* infection (22). Next, to investigate the role of NLRP6 in sepsis, we induced moderate sepsis in NLRP6 KO and WT mice through CLP. The KO mice had better survival (80% vs 40%) compared to that of WT mice (**Figure 1A**). To examine whether the resistant phenotype in the KO mice is model specific, we infected KO and WT mice with a sub-lethal dose of *E. coli* intraperitoneally to induce bacterial sepsis. Consistent with the CLP model, the KO mice had improved survival compared to that of WT mice during *E coli*-induced sepsis (**Figure 1B**). Furthermore, we measured bacterial burden in multiple tissues, including spleen, kidney, and PF following sepsis. The KO mice had less bacterial burden in the PF, spleen, and kidney compared to that of WT mice at both 12- and 24-hours post-sepsis (**Figures 1C-E**). In previous studies, the NLRP6 inflammasome has been implicated in regulating gut microbiota composition (18, 20). To determine whether differences in gut microbiota can modulate the outcome of sepsis, we co-housed WT and KO mice for 4 weeks and induced sepsis. The co-housed KO mice still had less bacterial burden in the PF, spleen, and kidney compared to that of WT mice, indicating that the phenotype observed in the KO mice is not microbiota dependent (**Figures 1F-H**).

### NLRP6 augments neutrophil recruitment and cytokine production during sepsis

**Figure 2:**
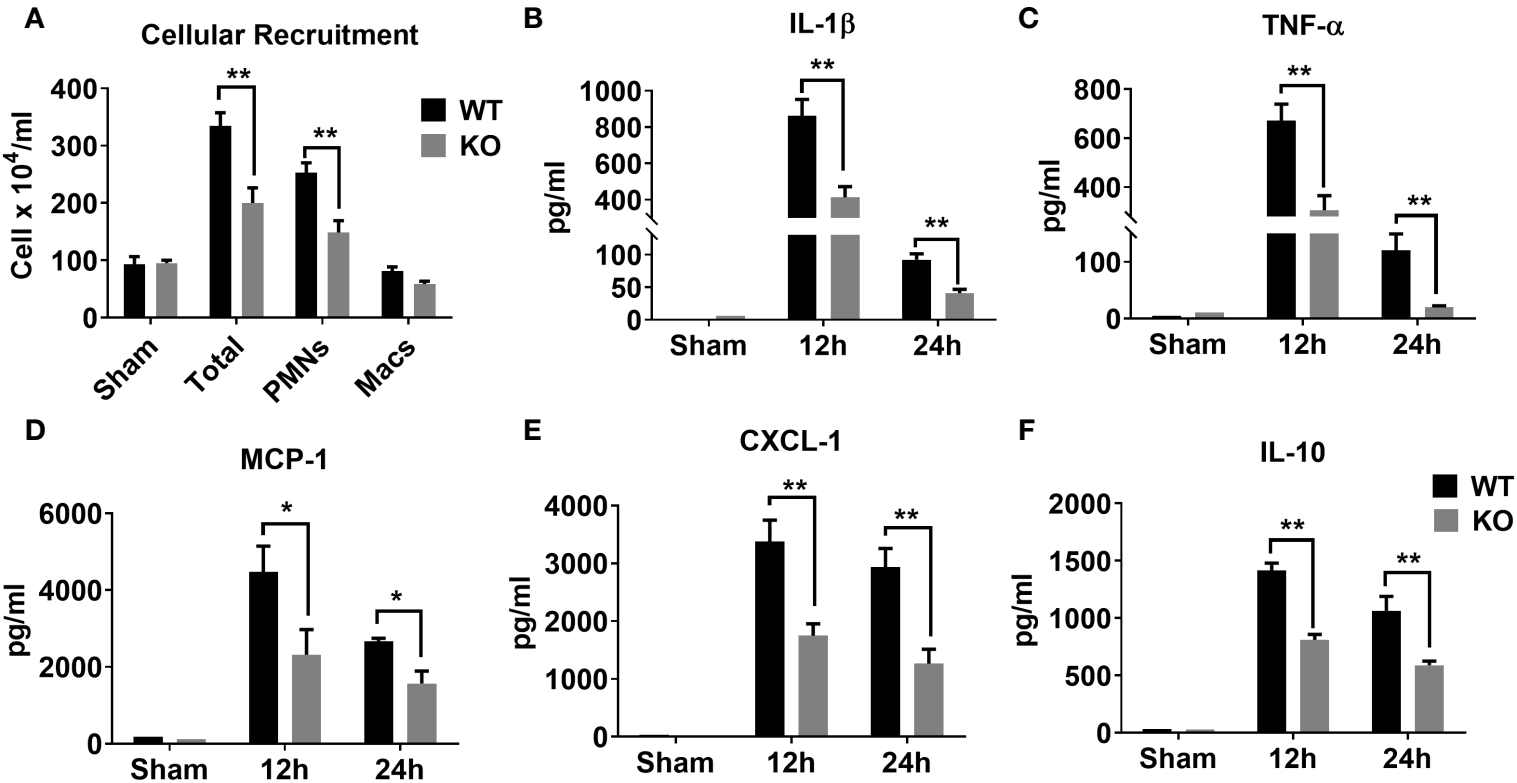
Role of NLRP6 in inflammation after CLP-induced sepsis. **(A)** CLP- and sham operated WT and NLRP6 KO mice (N=6-10 per group) were euthanized at designated time points to collect PF. Total and differential cell counting were performed in PF using Diff-Quik staining and light microscopy. Data were analyzed using Multiple *t* tests: one per row (*p<0.01). **(B)** IL-1β, **(C)** TNF-α, **(D)** MCP-1, **(E)** CXCL-1, and **(F)** IL-10 were measured in PF using standard ELISA procedure. All data are represented as Mean ± SEM. Data were analyzed using unpaired two-tailed student’s *t* test for each time point (*p<0.05 and **p<0.01). CLP, Cecal ligation and puncture, PF, Peritoneal lavage fluid, IL-1β, Interleukin-beta, TNF-α, Tumor necrosis factor-alpha, MCP-1, Monocyte chemoattractant protein-1, CXCL-1, C-X-C motif chemokine ligand 1, and IL-10, Interleukin-10.

Sepsis is characterized by an initial hyper-inflammatory phase followed by a prolonged hypo-inflammatory or immunosuppressive phase (27, 28). Both of these phases are implicated in death of septic patients. Therefore, we determined whether NLRP6 initiates hyperinflammation to enhance mortality during sepsis. To this end, we induced sepsis through CLP and measured inflammatory cell recruitment in the PF at 24 hrs. Interestingly, WT mice had more total WBCs and neutrophils recruited in the peritoneal cavity compared to that of KO mice after sepsis (**Figure 2A**). In addition, we measured major proinflammatory cytokines in the cell free PF obtained from septic mice. The WT mice had increased levels of IL-1β, TNF-α, MCP-1, and CXCL1 compared to that of KO mice (**Figures 2B-E**). Reduction in IL-1β in the PF of KO mice compared to WT mice indicates that the NLRP6 inflammasome is activated during sepsis. Moreover, IL-10, which is considered predictive of fatal outcome in sepsis (29, 30), was also more abundant in WT compared to that of KO at 24 hours post-CLP (**Figure 2F**).

### Increased NLRP6 expression in T lymphocytes of septic patients and septic mice

**Figure 3:**
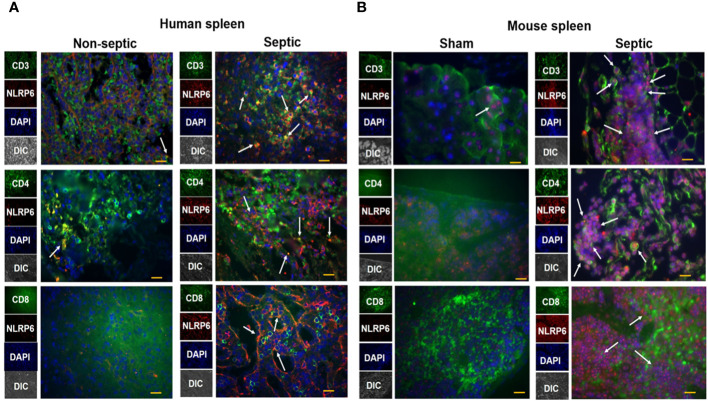
Expression of NLRP6 in lymphocytes of septic human patients and septic mice following CLP. **(A)** Tissue sections from septic and non-septic patients were processed for immunofluorescence assay. Tissue was stained against anti-human CD3, CD4, CD8 T cells (Green), anti-human NLRP6 (Red), and DAPI (Blue). The white arrowheads represent NLRP6^+^ cells. **(B)** C57BL/6 wild type (WT) were either subjected to CLP or sham (control) surgery. Twenty-four hours post-CLP, mice were euthanized to collect spleen for histopathological processing. Paraffin embedded spleen sections were processed for immunofluorescence microscopy. Antibodies used were anti-mouse CD3, CD4, CD8 T cells (Green), anti-mouse NLRP6 (Red), and DAPI (Blue). The white arrowheads represent NLRP6^+^ cells. Each image is a representative image from 5 different fields. Magnification: 40X.

The spleen is an important organ which plays a critical role in combating infections during sepsis. To explore whether NLRP6 expression is upregulated in the spleen of septic patients, we obtained spleen tissue sections from septic and non-septic patients and performed immunofluorescence microscopy to determine the upregulation of NLRP6 in these tissue sections. NLRP6 (Red) was upregulated in CD3, CD4, and CD8 T cells (Green) of septic patients compared to that of non-septic patients (**Figure 3A**). Consistent with this finding, NLRP6 (Red) was upregulated in splenic CD3, CD4, and CD8 T cells (Green) of CLP-induced septic mice compared to sham-operated mice (**Figure 3B**).

### NLRP6 enhances sepsis-induced T lymphocytic death in the spleen

**Figure 4:**
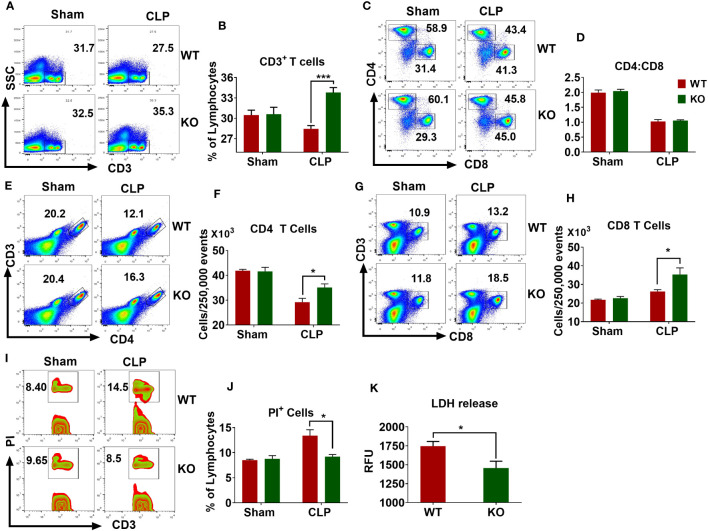
Importance of NLRP6 in sepsis-induced lymphocytic cell death in the spleen. WT and KO mice (N=6-8 per group) were subjected to CLP-induced sepsis. At designated time points, mice were sacrificed to collect spleen. The single cell suspensions obtained from septic and sham spleen were stained with appropriated fluorochrome-tagged antibodies and subjected to flow cytometric analysis. **(A)** A representative pseudo-color plot showing total CD3^+^ T cells. **(B)** Quantification of **(A)** **(C)** Representative figure showing the proportion of CD4 and CD8 T cells within CD3 cells. **(D)** Quantification of **(C)** **(E)** A flow cytometric figure showing CD3^+^CD4^+^ T cells. **(F)** Quantification of **(E)** **(G)** A representative flow cytometric figure displaying CD^+^CD8^+^ T cells. **(H)** Quantification of **(G)** **(I)** A representative zebra plot showing PI^+^CD3^+^ T cells. **(J)** Quantification of **(I)** All bar diagrams are expressed as Mean ± SEM. Figures **(B, F, H)**, and **(J)** were analyzed using unpaired two-tailed student’s *t* test (*p<0.05, and ***p<.001). **(K)** LDH release in the PF obtained from septic WT and KO mice (N=6-8 per group) was measured using an LDH assay kit. Data expressed as Mean ± SEM. Unpaired two-tailed student’s *t* test was used to analyze the data (*P<0.05). LDH=Lactate dehydrogenase.

Sepsis causes extensive loss of T-cells in the spleen that leads to immunosuppression and mortality (27, 31, 32). According to these observations, we examined whether the NLRP6 inflammasome regulates lymphocyte death during sepsis. For this, we first measured the number of T cells in the spleen after inducing sepsis and found that the WT mice had less CD3 positive lymphocytes compared to that of KO counterparts (**Figures 4A, B**). The normal CD4 to CD8 T cells ratio (2:1) observed in sham animals was reduced to 1:1 in both WT and KO mice after sepsis, suggesting that sepsis alters the proportion of CD4 and CD8 T cells in the spleen (**Figures 4C, D**). It is important to note that a similar observation was reported from the blood of septic patients (33). The number of CD4 T cells decreased after sepsis in both WT and KO mice; however, this reduction was less pronounced in the KO mice (**Figures 4E, F**). In contrast, the number of CD8 T cells increased in both WT and KO mice after sepsis; however, the increase was more pronounced in KO compared to that of WT counterparts (**Figures 4G, H**).

The reduced numbers of lymphocytes observed in the spleens of WT mice could be due to increased cell death. To this end, we induced sepsis in WT and KO mice and measured the extent of cell death in the spleen through flow cytometry. WT mice had more PI^+^ T cells compared to that of KO mice (**Figures 4I, J**). In addition, the PF obtained from septic KO mice displayed less LDH release compared to that of WT counterparts, indicating that NLRP6 enhances cell death during sepsis (**Figure 4K**).

### CD8 T cells but not CD4 T cells mediate bacterial clearance after sepsis

**Figure 5:**
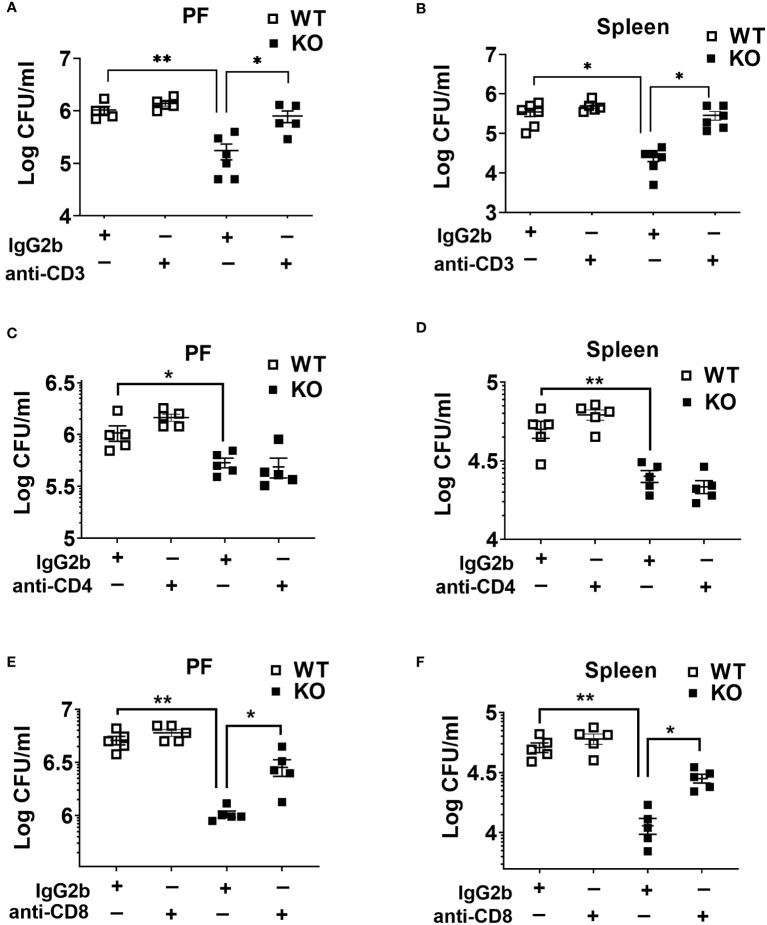
Role of T cells in host protection during CLP-induced sepsis. **(A)** and **(B)** WT and NLRP6 KO mice (N=5/group) were treated with either isotype antibody or anti-CD3 antibody prior to induction of CLP. Twenty-four hours post-CLP, mice were euthanized to measure bacterial burden in **(A)** peritoneal fluid (PF) and **(B)** Spleen. **(C)** and **(D)** Mice from WT and NLRP6 KO group (N=5/group) were administered with either isotype or anti-CD4 antibody before induction of sepsis. After 24 hours, bacterial loads in **(A)** PF and **(B)** spleen were measured. **(E)** and **(F)** WT and NLRP6 KO mice (N=5/group) were treated with either isotype antibody or anti-CD8 antibody prior to induction of CLP. Twenty-four hours post-CLP, mice were euthanized to measure bacterial burden in **(E)** peritoneal fluid (PF) and **(F)** Spleen. Data in each graph are represented as Mean ± SEM. Data were analyzed using one-way ANOVA followed by Tukey’s multiple comparison test (*p<0.05 and **p<0.01).

It has been reported that the loss and dysfunction of T cell responses contribute to an immunosuppressive or hyporesponsive state of leukocytes during sepsis (27, 34). Because we found higher T cell depletion in WT compared to KO mice counterparts, we sought to determine their role in bacterial clearance in sepsis. In this context, we depleted T lymphocytes using specific antibodies against CD3, CD4, and CD8 cells. Administration of anti-CD3 antibody markedly increased the bacterial burden in NLRP6 KO mice (**Figures 5A, B**). Although depletion of CD4 T cells did not affect bacterial clearance during sepsis (**Figures 5C, D**), the depletion of CD8 T cells lead to increased bacterial burden in the PF and spleen in the KO mice (**Figures 5E, F**). In order to determine the efficiency of Ab depletion, 12 hours after antibody administration, we obtained blood samples for flow cytometry to determine the expression of CD4+, CD8+, and CD3e+ T cells. Our findings show that these antibodies are extremely efficient in depleting the specific T cell populations (data not shown).

### NLRP6-mediated susceptibility to sepsis is IL-18 but not IL-1β dependent

**Figure 6:**
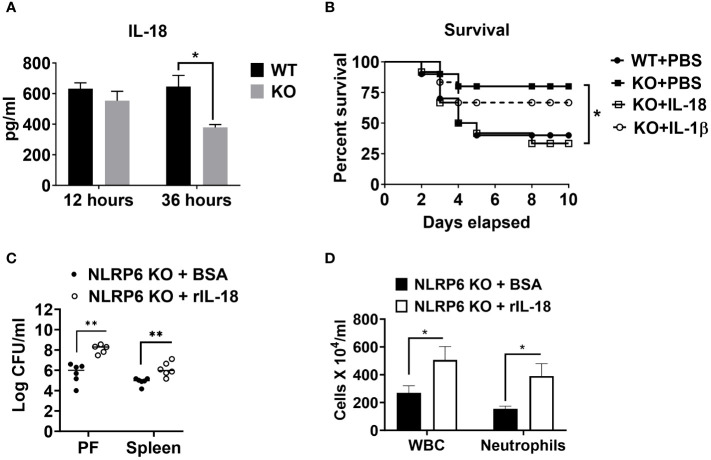
NLRP6-mediated enhanced susceptibility to CLP-induced sepsis is IL-18 dependent. **(A)** Sepsis was induced in WT and NLRP6 KO mice (N=6-8 per group) using CLP. At designated time points, mice were humanly euthanized to collect PF. IL-18 in cell free PF supernatants was measured using ELISA. Data are expressed as Mean ± SEM and were analyzed using unpaired two-tailed student’s *t* test (*p<0.05). **(B)** WT and NLRP6 KO mice (N=10 per group) were subjected to CLP-induced sepsis. WT mice received PBS right after CLP whereas, NLRP6 KO mice either received PBS, IL-18, or IL-1β immediately after CLP. Survival was monitored for 10 days (N=10 per group). The survival analysis was performed using Log-rank (Mantel-Cox) test (*p<0.05). **(C, D)** KO mice were subjected to CLP-induced sepsis. At 12-hours post-infection, mice were sacrificed to collect peritoneal lavage fluid (PF) and spleen to enumerate CFU **(C)** (N=5-6 mice/group) and obtain PF to enumerate total WBC and neutrophil recruitment **(D)** (N=3 mice per group). Data were analyzed using unpaired two-tailed student’s *t* test for each time point (*p<0.05, and **p<0.01).

Cytokines, such as IL-18 and IL-1β have been implicated in aggravating septic conditions (35–40). In addition, IL-1β and IL-18 are the two critical cytokines activated/cleaved by inflammasomes, including NLRP6 (15, 35). Therefore, we measured levels of IL-18 and IL-1β in PF of WT and KO mice and found that these cytokines were less abundant in KO mice compared to that of WT mice (**Figures 2B**, **6A**). Moreover, we hypothesized that reduced IL-18 and/or IL-1β in the KO mice could account for higher survival in these mice. To test this hypothesis, we administered recombinant IL-1β or IL-18 to the KO mice immediately after CLP and monitored their survival. IL-18 administration reversed the survival advantage observed in the KO mice following sepsis. However, no difference in the survival was observed in KO mice upon IL-β treatment, indicating that NLRP6 increases susceptibility via IL-18, but not through IL-1β (**Figure 6B**). In addition, recombinant IL-18 administration immediately following CLP augmented bacterial burden in PF and spleen (**Figure 6C**) as well as enhanced WBC and neutrophil recruitment to PF (**Figure 6D**).

### NLRP6-driven IL-18 elicits determinantal inflammation following sepsis

**Figure 7:**
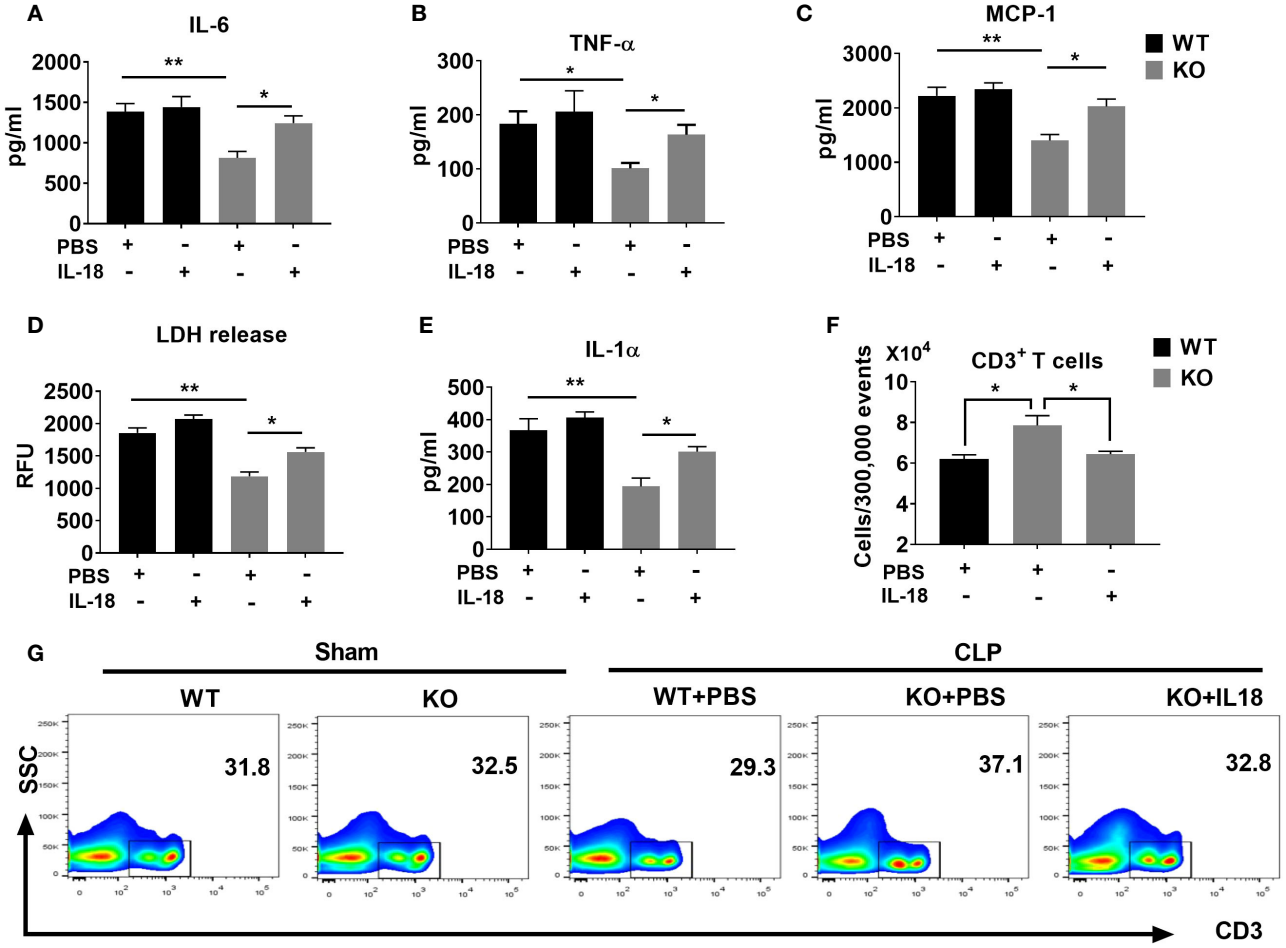
The NLRP6 inflammasome driven IL-18 elicits an adverse inflammatory response during CLP-induced sepsis. We induced sepsis in WT and NLRP6 KO mice (N=6-8 per group) through the CLP procedure. WT mice received PBS whereas the KO mice either received PBS or IL-18. At 36 hours post-CLP, mice were sacrificed to collect PF. **(A)** IL-6, **(B)** TNF-ἀ, and **(C)** MCP-1 were measured in the PF using ELISA. **(D)** LDH released in the PF was measured using a Fluorometric kit. **(E)** IL-1α was measured in PF using ELISA. Data are expressed as Mean ± SEM in each bar diagram. Data from **(A-F)** were analyzed using one-way ANOVA followed by Tukey’s multiple comparisons test (*p<0.05, and **p<0.01). **(G)** Spleens obtained from mice in A were processed to generate single cell suspensions. These cells were then stained with antibodies to estimate the numbers of T cell subpopulations using flow cytometry. **(F)** Absolute numbers of T cells per 300,000 events. **(G)** Representative plot showing percentage of CD3^+^ T cells.

Finally, we evaluated the mechanisms by which IL-18 enhances mortality in NLRP6 KO mice following sepsis. Since NLRP6 KO mice displayed attenuated inflammation and less T cell death, we hypothesized that IL-18 could contribute to hyper-inflammation and high lymphocyte loss in the WT mice ultimately leading to mortality. To this end, we administered recombinant IL-18 (rIL-18) to the KO mice immediately after sepsis and determined the degree of inflammation. Mice that received rIL-18 had higher levels of inflammatory cytokines, such as IL-6, TNF-α, and MCP-1 compared to the groups that did not receive IL-18 (**Figures 7A-C**). In addition, we measured the extent of cell death by determining the levels of cell death alarmins (LDH and IL-1α) (14) in the PF after administration of rIL-18. Interestingly, the KO group that received rIL-18 showed higher levels of alarmins in the peritoneal lavage fluid suggesting higher cell death compared with the KO mice group that received PBS (**Figures 7D, E**). Since the KO mice had less cell death, we measured the T cell population in the spleen after induction of sepsis. Intriguingly, the KO mice that received rIL-18 had less CD3^+^ T cells in the spleen compared to the group that received PBS only (**Figures 7F, G**).

## Discussion

Sepsis remains a leading cause of death in Intensive Care Units (ICUs) and is a complex disorder associated with multiple organ failure due to dysregulated host response to microbial insult (1, 3–5). Sepsis is characterized by a first phase of hyperinflammatory response followed by a second phase of immunosuppression both of which contribute to mortality. However, the mechanisms underlying the initiation of exaggerated inflammation and subsequent immunosuppression are not completely understood. Through this investigation, we have demonstrated that NLRP6 is upregulated in different lymphocytic cells of the spleens from septic patients and septic mice. Genetic disruption of NLRP6 enhances the survival during CLP-induced polymicrobial sepsis and *E. coli*-induced sepsis models. The NLRP6 inflammasome triggers a destructive inflammatory response and enhances sepsis-induced loss of lymphocytes. In addition, we have shown that NLRP6-driven IL-18 enhances mortality in sepsis through mediating both inflammation and cell death.

Multiple studies have identified NLRP6 as a regulator of host defense. Negative regulation of host defense by the NLRP6 inflammasome has been reported in inflammatory settings, including pulmonary (14) and systemic bacterial infections (15, 18). Deletion of NLRP6 was found to be beneficial for the host in these studies. Similarly, we and others have demonstrated the detrimental role of the NLRP3 inflammasome during sepsis (12, 13). Although these models have similar phenotypes, the mechanisms associated with these findings are model specific. In a systemic bacterial infection model, NLRP6 was found to regulate MAPK and canonical NF-kB pathway to enhance neutrophil recruitment and bacterial clearance (18). In a MRSA-induced pneumonia model, NLRP6 KO mice had higher neutrophil accumulation in the lungs due to reduced cell death (14). In both models, higher neutrophil recruitment was found to be important to clear bacteria from the organs. However, in the sepsis models, we found that the NLRP6 KO mice had reduced neutrophil recruitment, attenuated cytokine production, augmented bacterial clearance, and higher survival. In support of these findings, higher neutrophil recruitment along with excessive cytokine production have been shown to worsen the outcome of sepsis (29, 41, 42). In contrast to our finding, NLRP6 was found important to clear *C. rodentium* (20) and encephalomyocarditis viral infection from the intestine (21), indicating that NLRP6 is important to clear enteric pathogens. Furthermore, NLRP6 serves as a positive regulator of host defense in response to intrapulmonary *K. pneumoniae* infection (22). These observations suggest that the role of NLRP6 could be model or organ specific. In localized infection models such as intestinal and pulmonary inflammation where neutrophils are important to clear pathogens, NLRP6 could play a crucial role; however, in the septic model, NLRP6-induced inflammation is detrimental. This spatiotemporal response of NLRP6 has made this NLR unique among its family members.

Several studies have reported that the NLPR6 inflammasome regulates gut microbiota composition and such differences in microbiota composition render the mice susceptible to colitis and tumorigenesis (16, 18, 20, 23). However, recent studies have challenged these observations stating that the NLRP6 inflammasome do not shape the microbiota composition (43, 44). Although the NLRP6 inflammasome regulation of the microbiota composition is debatable, we confirmed that the resistant phenotype observed in the NLRP6 KO mice is not dependent on their microbiota composition. The co-housed KO mice displayed a similar phenotype as that of singly housed KO mice confirming that NLRP6 regulates sepsis independent of microbiota composition. Our findings are in agreement with previous studies that NLRP6 regulates host defense against bacterial infections independent of microbiota composition (14, 18).

In a mouse model of CLP-induced sepsis, it was reported that sepsis induces CD8+ T cell (memory and naïve) exhaustion which affects host defense (45). Loss of lymphocytes, particularly through apoptosis and inflammatory modes of cell death is a hallmark of sepsis-induced immunosuppression (31, 46, 47). Lymphocytes have been shown to play critical roles during sepsis (48, 49). Our study also confirmed this finding and further demonstrated that T lymphocytes, especially CD8 T cells are crucial to clear pathogens during sepsis. Blocking these cells using antibodies markedly increased the bacterial burden in the peritoneal cavity and the spleen.

Inflammasome activation leads to cleavage of IL-1β and IL-18 from the pro form to mature form. We have demonstrated that the NLRP6 inflammasome elicits hyperinflammation as evidenced by higher cytokine storm and augmented granulocyte recruitment. Furthermore, we have found that NLRP6 triggers hyperinflammation via IL-18. We found that NLRP6-driven IL-18 but not IL-1β contributes to sepsis-induced lymphocyte death, which were found to be crucial for host defense. This conclusion is further supported by the findings that addition of recombinant IL-18 abolished the survival advantage in the NLRP6 KO mice. Consistent with our findings, IL-18 has been shown to play detrimental roles in septic conditions (35–37, 50), including pneumonia-induced sepsis (51). In future studies, it is important to determine the cellular origin of IL-18 during sepsis because IL-18 is known to be produced by numerous cell types, including dendritic cells, T cells, macrophages, and epithelial cells and to activate immune cells, including T cells and NK cells through autocrine and paracrine loops (52–54).

Overall, the current study uncovered a previously unrecognized role of the NLRP6-IL-18 axis during sepsis. Using human and animal samples, we have demonstrated that NLRP6 is upregulated in multiple cell types, including CD4 and CD8 T cells in the spleen during sepsis. Furthermore, NLRP6 plays a detrimental role in host defense during sepsis through IL-18-mediated destructive inflammation. Based on these results, we propose that blocking NLRP6 could be an effective therapy to reduce sepsis-related mortality.

## Data availability statement

The original contributions presented in the study are included in the article/supplementary material. Further inquiries can be directed to the corresponding author.

## Ethics statement

The animal study was approved by Louisiana State University and A&M College-Baton Rouge. The study was conducted in accordance with the institutional requirements.

## Author contributions

Conceived and designed experiments: LG and SJ. Performed experiments: LG, SP, JL, LJ, SC, and DB. Analyzed data: LG and SJ. Wrote the paper: LG and SJ. All authors contributed to the article and approved the submitted version.
